# Recent advances in microwave-assisted dry reforming of methane: catalyst, performance, and fundamentals

**DOI:** 10.3389/fchem.2025.1681282

**Published:** 2025-09-17

**Authors:** Siyu Kuang

**Affiliations:** State Key Laboratory of Chemical Safety, SINOPEC Research Institute of Safety Engineering Co., Ltd., Qingdao, Shandong, China

**Keywords:** dry reforming of methane, syngas, catalysts, microwave-assisted, sintering

## Abstract

The dry reforming of methane converts methane and carbon dioxide into syngas (a mixture of H_2_ and CO), which can be utilized for synthesizing downstream chemical products. However, its high endothermicity necessitates elevated operating temperatures (∼900 °C), posing challenges in energy efficiency and catalyst stability. Microwave-assisted heating has emerged as a promising alternative to conventional thermal catalysis, offering potential for enhanced reaction rates, improved energy utilization, and catalyst reactivation. This review systematically examines the recent advancements in microwave-assisted dry reforming of methane. It begins with an analysis of the reaction thermodynamics and fundamentals of microwave heating, specifically addressing its mechanisms and advantages over conventional methods. The core of the review focuses on the rational design of catalysts tailored for effective microwave absorption and catalytic performance. A critical comparison of catalyst performance under microwave *versus* conventional heating is provided, highlighting the roles of microwave in boosting conversion, suppressing coke deposition, and enhancing catalyst longevity. Finally, the review discusses the persistent challenges in scaling this technology and proposes future research directions, particularly in catalyst and reactor design and process intensification. This work underscores the transformative potential of microwave catalysis to drive efficient and sustainable dry reforming of methane processes.

## 1 Introduction

Over the past decade, with the rapid growth of the world’s population, the consumption of resources has also shown a rapid increasing trend (Li, 2005). During the rapid industrialization process, the increasing energy demand has gradually changed the energy structure (Tanksale et al., 2010). At present, the world’s energy sources are still dominated by the three traditional fossil fuels: coal, oil and natural gas. The consumption of fossil energy generates a large amount of greenhouse gases and lead to serious environmental problems (Abdullah et al., 2017). Carbon dioxide (CO_2_) is one of the typical greenhouse gases and a major contributor to the greenhouse effect. But at the same time, it is also a natural source of carbon (Markewitz et al., 2012; Sokolov et al., 2012). In recent years, due to the abundant reserves of shale gas and the discovery of improved hydraulic fracturing technology, methane (CH_4_) has become a preferred energy source (Olah et al., 2013; Olah et al., 2015). However, a significant portion of natural gas, particularly associated gas from oil extraction, is inefficiently utilized because of technical, regulatory, or economic constraints. The World Bank’s Global Gas Flaring Reduction Partnership (GGFR) estimates that ∼143.4 billion cubic metres (b.c.m.) of associated petroleum gas (APG), equalling a sales value of US$16.5 billion, has been burned in 2021 (Elvidge et al., 2018; Liu et al., 2023). In this case, the reduction of carbon dioxide and methane emissions and their reuse are extremely urgent. Under the background of global warming, Carbon Capture Utilization and Storage (CCUS) is currently one of the most important technical options for achieving the low-carbon utilization of fossil energy (Conti et al., 2016). Within the framework of CCUS, the utilization methods of CO_2_ mainly include mineral carbonization, physical utilization, chemical utilization and biological utilization, etc. In recent years, oriented towards the production of valuable chemical products using carbon dioxide as raw material (Kondratenko et al., 2013), a large number of scientific research efforts have been dedicated to developing carbon dioxide reuse technologies, mainly including reforming reactions, hydrogen addition reactions, photoreduction reactions, electroreduction reactions, and copolymerization reactions (Huang et al., 2024; Saeidi and Amin, 2014; Wang et al., 2025; Yu et al., 2008).

Among the above-mentioned carbon dioxide reuse technologies, the methane-carbon dioxide reforming reaction provides an effective approach for converting carbon dioxide and methane and preparing syngas (H_2_ and CO). The syngas produced by the methane-carbon dioxide reforming reaction has a relatively low molar ratio of H_2_/CO (H_2_/CO = 1), and can be used as a general raw material for the synthesis of downstream high value-added chemical products (acetic acid (Schwab et al., 2015), dimethyl ether (Azizi et al., 2014), long-chain hydrocarbons (Gadalla and Bower, 1988; Mo et al., 2014), etc.). Therefore, the methane-carbon dioxide reforming reaction can not only convert and utilize carbon dioxide, but also provide a promising indirect utilization route of methane. This technology is of great significance for the efficient utilization of abundant natural gas resources, as well as for reducing carbon dioxide emissions and alleviating the greenhouse effect. Despite its economic and environmental potential, the dry reforming of methane (DRM) remains an immature industrial process, primarily due to challenges in catalyst development (Das et al., 2019; Park et al., 2019). The major issue is catalyst deactivation, which occurs through coke and carbon formation on the catalyst surface and through thermal sintering (Bradford and VANNICE, 1999; Mort et al., 2015; Pakhare and Spivey, 2014). Since the DRM reaction is highly endothermic and requires high reaction temperatures (above 700 °C), it accelerates both catalyst thermal sintering and the formation of local “cold spots” within the catalyst bed (Chen et al., 2012). Therefore, effective heat transfer control is crucial for the successful implementation of DRM.

Furthermore, the significant thermal energy demand of the high-temperature DRM reaction presents a major economic hurdle for standalone application. However, this challenge also unveils a significant opportunity for integration with other industrial processes. Many energy-intensive industries (e.g., metallurgy, cement production, chemical synthesis) generate vast amounts of high-grade waste heat, which often remains underutilized (Li, 2017). Coupling the endothermic DRM process with such sources of waste heat could dramatically improve its overall energy efficiency and economic attractiveness, transforming an industrial liability into a valuable resource for chemical production and emissions mitigation (Jastrząb, 2018; Liao and Horng, 2017; Tan et al., 2025). This synergy between waste heat recovery and catalytic CO_2_ utilization represents a promising direction for sustainable process intensification.

Compared to conventional heating, microwave heating offers several advantages, such as higher heating rates, better heating control, reduced equipment size, and significant time and energy savings (Bao et al., 2023; Jie et al., 2020; Jones et al., 2002; Motasemi, 2013). Microwave-assisted heating has been widely used to activate environmental catalytic reactions and catalytic deconstruction of plastic waste into hydrogen and high-value carbons (Bao et al., 2023; Jie et al., 2020). The application of microwave-assisted technology as a novel approach for DRM was first introduced by Shah and Gardner (Shah and Gardner, 2014). Microwave-assisted methane reforming has attracted significant attention over the past 2 decades due to its markedly higher conversion rates and yields, as well as reduced coke deposition, compared to conventional reforming systems (la Hoz et al., 2005; Jacob et al., 1995; Berlan, 1995). This technology is poised to play a key role in the near future by enabling the conversion of offshore and remote natural gas into liquid fuels via compact-to medium-scale reactors, using syngas (a mixture of H_2_ and CO) as a key intermediate (Hwang et al., 2013; Kim et al., 2014). There are presently several reviews available on the development of this field (Fidalgo et al., 2008; Nguyen Sunars et al., 2020; Palma et al., 2020; Zha et al., 2023). Nevertheless, the existing reviews have not exhaustively covered all emerging aspects and recent advances in this rapidly evolving field, such as new catalyst design systems and comparisons of energy consumption with conventional heating. Therefore, the aim of this paper is to provide a comprehensive review of catalysts and their catalytic performance in microwave-assisted DRM, with a particular focus on the challenges and opportunities associated with this process.

## 2 Overview

### 2.1 Brief history

As a reforming technology with commercial potential, DRM technology has become a research hotspot for researchers in various countries in recent years. In 1928, Fisher and Tropsch first proposed the concept of DRM (Fisher, 1928), with the aim of converting methane into syngas. Since then, related literature on DRM have been published one after another. It was not until the 1990s, affected by global climate warming, that carbon dioxide emission reduction became a common problem faced by the whole world. At this time, DRM technology gradually attracted widespread attention (Ashcroft et al., 1991; Gree et al., 1992).

DRM (Equation 1) is an extremely endothermic reaction:

$$CH_{4}+CO_{2}\to 2CO+2H_{2},∆H_{298K}^{0}=247\;kJ/mol$$ (1)

In addition, the DRM is a complex reaction system (Table 1). Usually, in addition to the main reaction, there are also side reactions such as the revise water-gas shift reaction (RWGS), methane cracking reaction, and carbon monoxide disproportionation (Boudouard reaction) (Fan and Bhatia, 2009). It can be known from Equation 1 that DRM is a strong endothermic reaction in thermodynamics (∆H_298K_ = 247 kJ/mol), and it requires a relatively high temperature to proceed. According to the thermodynamic calculation results, it is known that the minimum temperature required for the reforming reaction between methane and carbon dioxide is 640 °C, and increasing the temperature is conducive to the forward progress of the reaction (Wang, 1996). Usually, the use of effective catalysts can lower the reaction temperature. But even so, the high reaction temperatures mean that the catalyst needs to have good thermal stability, which is undoubtedly a strict requirement.

**TABLE 1:** Different reactions during DRM (Nikoo and Amin, 2011).

Reactions	ΔH_298K_ kJ/mol	Ln (K_eq_) at 573K	Ln (K_eq_) at 1373K	Temperature where reaction occurs
CH_4_ + CO_2_ ⇌ 2CO + 2H_2_	247	−20	13	>973K
CO_2_ + H_2_ ⇌ CO + H_2_O	41	−5	2	>973K
2CH_4_ + 2CO_2_ ⇌ C_2_H_4_ +2CO + 2H_2_O	284	−36	0	>1473K
C_2_H_6_ ⇌ C_2_H_4_ + H_2_	136	−14	4	>1073K
2CH_4_ + CO_2_ ⇌ C_2_H_6_ + CO + H_2_O	106	−19	−5	>1473K
CO_2_ + 3H_2_ ⇌ CH_3_OH + H_2_O	−49.1	−12	−20	<573K
CO + 2H_2_ ⇌ CH_3_OH	−90.6	−10	−20	<573K
2CO ⇌ C + CO_2_	−172.4	15	−7	<973K
CH_4_ ⇌ C + 2H_2_	74.9	−6	5	>873K
H_2_ + CO ⇌ H_2_O+ C	−131.3	12	−6	<873K
CO_2_ + 2H_2_ ⇌ C + 2H_2_O	−90	8	−5	<973K
3H_2_O+ CH_3_OCH_3_ ⇌ 2CO_2_ + 6H_2_	136	20	37	>573K
CH_3_OCH_3_ + CO_2_ ⇌ 3CO + 3H_2_	258.4	10	40	>573K
2CH_3_OH ⇌ CH_3_OCH_3_ + H_2_O	−37	3	−1	<973K
CH_3_OCH_3_ + H_2_O ⇌ 2CO + 4H_2_	204.8	14	37	>573K
CO + 3H_2_ ⇌ CH_4_ + H_2_O	−206.2	14	−11	<873K
CO_2_ + 4H_2_ ⇌ CH_4_ + 2H_2_O	−165	14	−10	<873K

Furthermore, the reaction equilibrium of DRM for syngas production is usually affected by the concurrent reverse water gas shift (RWGS) reaction. This leads to the CO_2_ conversion rate usually being higher than the CH_4_ conversion rate at equilibrium, thereby resulting in the generated syngas H_2_/CO being lower than 1 (Gadalla and Bower, 1988). Apart from the influence of the side reaction RWGS, the formation of carbon deposits is the main cause of the inactivation of the catalyst during the DRM process. The formation of carbon deposits during the DRM reaction is mainly attributed to two side reactions: CH_4_ cracking and CO disproportionation (Jang et al., 2016). Although raising the temperature and increasing the amount of CO_2_ feed can reduce carbon deposits, both of these pose certain problems in industrial production. Excessively high reaction temperatures impose strict requirements on reforming equipment, while excessive CO_2_ feedstock gas will consume a large amount of heat. Therefore, from the perspective of industrial production, conducting the reforming reaction at a lower temperature and with a smaller stoichiometric ratio of CH_4_/CO_2_ is a more appropriate choice.

**FIGURE 1:**
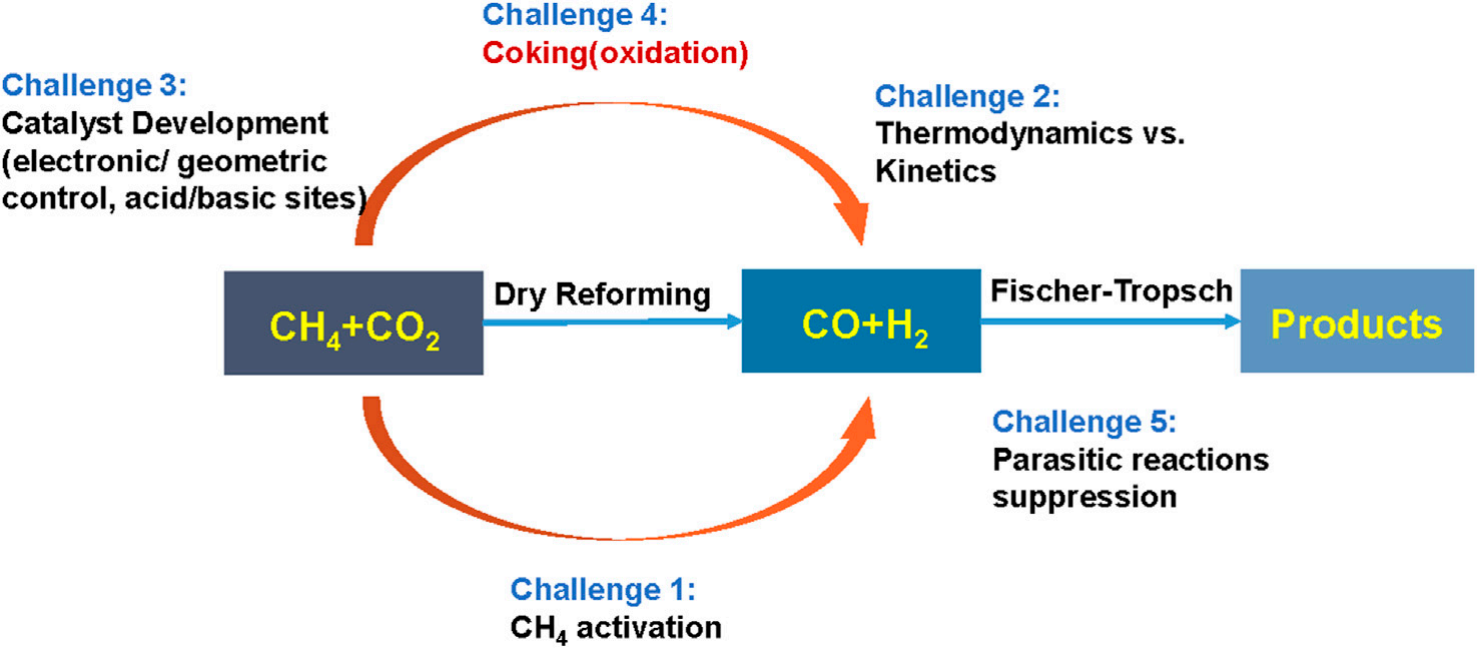
Challenges in DRM reaction. Adapted from Ref (Hussien and Polychronopoulou, 2022).

Although the DRM reaction has considerable potential for environmental friendliness, it is highly endothermic and carbon deposits form too rapidly during the reaction process, ultimately leading to the rapid deactivation of the catalyst (Figure 1). Therefore, the DRM reaction is not considered an industrially mature process.

### 2.2 Thermodynamics

The DRM reaction is a strongly endothermic reaction. Since both CH_4_ and CO_2_ are very stable molecules, their dissociation energies are 435 (CH_3_-H) and 526 (CO-O) kJ/mol, respectively. Therefore, a relatively high temperature is required to achieve the equilibrium conversion to syngas. On the other hand, the reaction equilibrium is also affected by the reverse water-gas shift side reaction (RWGS, Equation 2).

$$CO_{2}+H_{2}\to CO+H_{2}O,∆H_{298K}^{0}=41\;kJ/mol$$ (2)

This reaction leads to a CO_2_ conversion rate higher than the CH_4_ conversion rate at equilibrium. In practice, this is beneficial for the production of syngas with a H_2_/CO ratio of one or lower.

Apart from the RWGS reaction, in the DRM process, the formation of carbon deposits is also a major cause of catalyst deactivation. The carbon formed during the DRM process is mainly attributed to two reactions (Equations 3, 4):

$$CH_{4}\to C+{2H}_{2},∆H_{298K}^{0}=75\;kJ/mol$$ (3)

$$2CO\to C+{CO}_{2},∆H_{298K}^{0}=-172\;kJ/mol$$ (4)

Wang et al. reported that the decomposition of CH_4_ occurs above 557 °C, while the disproportionation reaction of CO occurs below 700 °C (Wang, 1996). To consider the conversion rates of CH_4_ and CO_2_ and the formation of carbon deposits, the optimal reaction temperature when the feed ratio CH_4_/CO_2_ = 1:1 is usually 870 °C–1,040 °C. In addition, some researchers have conducted systematic thermodynamic simulations on the effects of various experimental conditions such as reaction temperatures, CH_4_/CO_2_ feed ratio, reaction pressure, and additional oxidants on the DRM reaction, and have considered various reactions that lead to the formation of carbon deposits (Jang et al., 2016; Nikoo and Amin, 2011). They generally believe that the DRM reaction being carried out under high temperature and low-pressure conditions above 850 °C is necessary to achieve high conversion rates of CH_4_ and CO_2_.

## 3 Microwave heating technology

A new technique that received extensive attention in the last decades is based on using microwave energy potential for reforming of methane and carbon dioxide. In comparison to conventional heating methods, microwave applications for producing the required heat for reaction is more energy efficient and less expensive. Utilizing microwaves also provides more advantages than conventional heating including a rapid process heating, reduced processing time and work space, more accurate and uniform heating, and high quality (la Hoz et al., 2005; Caddick and Fitzmaurice, 2009; Ku et al., 2002; Gude et al., 2013).

Microwaves are electromagnetic waves whose wavelengths span 1 m–1 mm, corresponding to frequencies of 0.3–300 GHz. For practical heating purposes, the industrial, scientific and medical (ISM) bands—most commonly 0.915 GHz and 2.45 GHz—are employed to avoid interference with telecommunication services (Meredith, 1998). Whereas conventional heating relies on conduction, convection and radiation from a hot medium, microwave heating originates from the direct interaction of polar or conducting species with the oscillating electric (and, to a lesser extent, magnetic) field.

Currently, microwave heating has been widely applied in various fields, such as industrial wood drying, food processing, and rubber vulcanization, *etc.* Moreover, it has also been studied in areas like ceramic and polymer processing (Hoogenboom and Schubert, 2007; Komarneni et al., 1992; Wiesbrock et al., 2004), environmental applications (Jones et al., 2002; Verma and Samanta, 2018; Zhang and Hayward, 2006; Zlotorzy nski, 1995), biofuels and chemical production (Aravind et al., 2020; Hassan et al., 2020), as well as metallurgy and mineral processing (Jones et al., 2002; Kingman et al., 2004). It is worth noting that microwave heating technology has also been extensively studied in heterogeneous gas-phase catalysis (Durka et al., 2009; Zhang and Hayward, 2006), such as ammonia decomposition (Guler et al., 2017), methane decomposition (Domínguez et al., 2007; Zhang et al., 2003), hydrogen sulfide decomposition (Xu et al., 2017), nitrogen oxide and sulfur dioxide reduction (Peng et al., 2017; Zhang et al., 2001), CO_2_ reforming of CH_4_ (Lim and Chun, 2017; Zhang et al., 2003), and recently, alcohol steam reforming (Durka et al., 2011; Gündüz, 2015; Sarıyer et al., 2019). As for CO_2_ reforming of CH_4_, under microwave irradiation, CH_4_ and CO_2_ conversions regularly exceed thermodynamic equilibrium values predicted for the bulk temperature, while coke deposition is suppressed (Fidalgo et al., 2008; Fidalgo and Menéndez, 2012). The origin of this enhancement lies in the formation of microscopic “hot spots” on catalytic surfaces where local temperatures can be 100–300 K above the surrounding gas, accelerating both C–H and C=O bond scission (Fidalgo et al., 2008). Thus, microwave-assisted DRM offers a low-carbon, energy-efficient route to syngas (H_2_/CO) with markedly reduced external heat demand and a smaller reactor envelope compared to conventionally heated fixed-bed or fluidised-bed systems.

**FIGURE 2:**
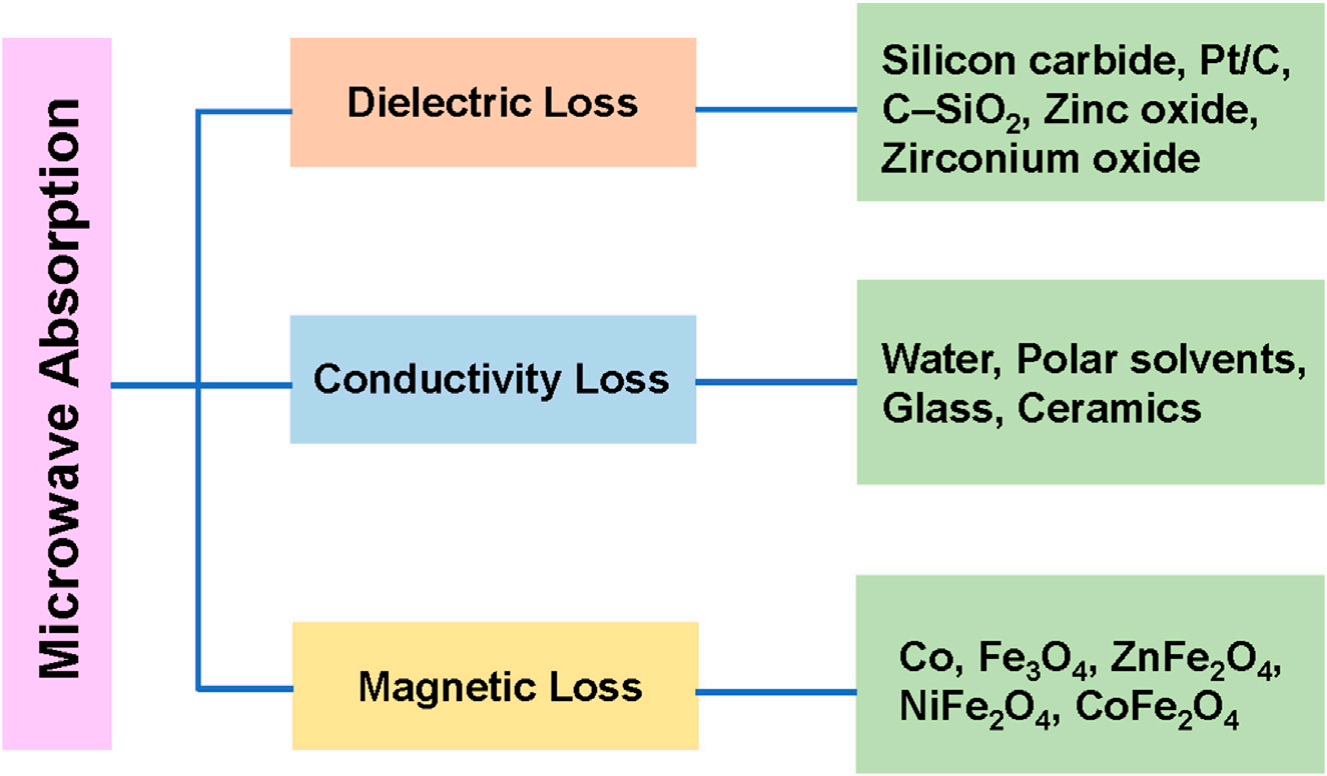
Summarized mechanism of microwave losses.

In fact, both components of the high-frequency electromagnetic radiation contribute to microwave heating (Figure 2). However, the dielectric heating caused by the electric field component is usually used to represent microwave heating, while the heating effect caused by the magnetic field component is rarely mentioned due to a lack of knowledge (Sun et al., 2016). When polarized molecules attempt to align with the high-frequency electric field by rotating, they collide with each other, resulting in dielectric heating. Therefore, the heating capacity of dielectric can be described by its dielectric loss tangent (tan δ_ε_), as shown in Equation 5:

$$\tan\delta_{\varepsilon }=\varepsilon ’’/\varepsilon ’$$ (5)

The real (ε′) and imaginary (ε″) components of complex permittivity quantify, respectively, how readily microwaves penetrate a material and how efficiently that material converts the incident electromagnetic energy into heat (Jones et al., 2002; Menéndez et al., 2010). A widely cited criterion for effective microwave absorption is a loss tangent (tan δ = ε″/ε′) exceeding 0.1 (Rossi et al.). The dielectric response of a specific sample is governed by its anisotropy, homogeneity, surface roughness, and critically by temperature and excitation frequency (Janezic, 2001). Ignoring the effect of factors related to its nature and the applied frequency, the loss tangent of a sample generally increases with its temperature at a frequency of 900 MHz or 2.45 GHz (Grant et al., 1998; Zhang and Hayward, 2002).

At room temperature, many solid materials such as alumina and silica (Nightingale, 2001) are classified as ultra-low-loss dielectrics. However, once a threshold temperature is surpassed, their heating ability rises sharply, as illustrated in Table 2. Moderately lossy composites such as carbon-coated SiO_2_ (C–SiO_2_) or platinum-decorated carbon (Pt/C) exhibit an even more pronounced effect, with tan δ doubling above 800 °C relative to ambient conditions. Such behaviour renders them particularly attractive for microwave-assisted DRM, where the elevated reaction temperature itself reinforces microwave absorption and sustains the required thermal profile (Zhang et al., 2018a). Consequently, a precise knowledge of the temperature-dependent dielectric behaviour of both catalyst and support is indispensable for the rational design of microwave-assisted DRM systems.

**TABLE 2:** Dielectric loss tangent (tan δ) of microwave receptor.

Material	Temperature (°C)	Frequency (GHz)	Dielectric properties			References
			ε′	ε″	tan δ	
Silicon carbide	25	3	N/A	N/A	0.58	Westphal, 2025
	20	2.45	13.7	6	0.437	Meredith (1998)
Pt/C	20	2.54	N/A	N/A	<0.27	Gangurde et al. (2017)
	850	2.54	N/A	N/A	<0.4	
C–SiO_2_	25	2.54	13.7	6	0.437	Hamzehlouia et al. (2018)
	>800	2.54	<15	12	>0.8	
Zinc oxide	25	2.54	3	3	1	Durka et al. (2009)
Zirconium oxide	25	2.54	20	2	0.1	

*N/A = not available in the reference.

In conductive materials, a high-frequency electric field induces an electric current, generating heat through collisions between charged particles and adjacent atoms or molecules (Meredith, 1998). While such conductive losses prevail in conductors, dielectric losses are dominant in lossy dielectrics like water, polar solvents, glass, and ceramics (Table 3). Nevertheless, in certain scenarios—such as microwave heating of electrolyte solutions (Horikoshi et al., 2012) or carbon-based materials (Menéndez et al., 2010)—both mechanisms contribute synergistically to heating within the high-frequency electric field, leading to an accelerated heating rate.

**TABLE 3:** Dielectric loss tangent (tan δ) of microwave transparent.

Material	Temperature (°C)	Frequency (GHz)	Dielectric properties			References
			ε′	ε″	tan δ	
Silicon	25	1	4.3	<0.05	<0.0116	Meredith (1998)
Alumina	590	2.54	10.37	0.027	0.0026	Bhattacharya and Basak (2016)
	980	2.54	11.06	0.15	0.01356	
	1,340	2.54	11.64	0.74	0.06412	
Fused silica	25	5.5	N/A	N/A	<0.0003	Durka et al. (2009)
	1,165	5.5	N/A	N/A	0.01	
Glass	25	3	4.82	0.026	0.0054	Durka et al. (2009)

*N/A = not available in the reference.

Existing experimental evidence indicates that microwave magnetic heating has advantages over electric heating in the heating of certain ferrites and conductive powders (such as Fe_3_O_4_, WC, Fe, and Co.) (Cheng and Agrawal, 2002; Cheng et al., 2001). Roy et al. reported that through a microwave-assisted synthesis method, new crystal structures of ferrites were prepared, including ZnFe_2_O_4_, NiFe_2_O_4_, BaFe_12_O_19_, CoFe_2_O_4_ and Fe_3_O_4_, etc (Roy et al., 2002). Moreover, it was found that the significant changes in the structural phase and magnetic properties of these ferrites were mainly attributed to the magnetic components. Collectively, existing studies confirm that magnetic losses play a substantial role in the microwave heating not only of magnetic materials, but also of conductors, semiconductors, and composites (Table 4) (Bhattacharya and Basak, 2016) (Cao et al., 2010; Haneishi et al., 2017; Rosa et al., 2016; Wen et al., 2011).

**TABLE 4:** Magnetic properties of some ferrites at 25 °C and 2.45 GHz.

Material	Magnetic properties		
	ε′	ε″	tan δ
NiZnFe_4_O_4_	1.080	0.201	0.186
CuZnFe_4_O_4_	1.038	0.209	0.201
BaFe_12_O_19_	1.105	0.071	0.064
CuFe_2_O_4_	1.029	0.106	0.103
Fe_3_O_4_	1.51	0.3	0.199

The magnetic heating ability of a material is described by its magnetic loss tangent (tan δ_μ_) as given by Equation 6:

$$\tan\delta_{\mu }=\mu ’’/\mu ’$$ (6)

The real (μ’) and imaginary (μ’’) parts of permeability represent the amount of energy stored and lost, respectively (Durka et al., 2009; Sun et al., 2016).

Due to the fact that low-lossy or transparent materials cannot be rapidly heated to the high temperature required for efficient dry reforming of methane reactions. Currently, the gas reactants in DRM process and most common catalysts, such as nickel-based catalysts loaded on SiO_2_, Al_2_O_3_ or other mesoporous materials (Abdullah et al., 2017; Lovell et al., 2015; Wang et al., 2018), exhibit relatively low microwave absorption due to their low dielectric loss tangent (Fidalgo et al., 2011). Therefore, it is necessary to develop catalysts for the microwave-assisted DRM process.

## 4 Catalysts for microwave-assisted DRM

Since DRM reaction takes place under the presence of a catalyst, and gaseous substances themselves possess intrinsic dielectric properties that are not appreciably favouring the microwave interaction (Hamzehlouia et al., 2018). Therefore, the exploration and development of microwave-assisted DRM reactions have mainly focused on solid catalyst materials. While the active metal sites (e.g., Ni, Fe, Pt, Ru) remain crucial for the surface reactions (Abdullah et al., 2017), the catalyst must now also perform as an efficient microwave receptor. By converting the absorbed microwave radiation into thermal energy and thereby serving as a heat source, microwave-assisted DRM can be achieved (Fidalgo et al., 2008; Hamzehlouia et al., 2018). Therefore, the development of microwave-assisted DRM catalysts not only requires considering the catalyst's ability to resist carbon deposition and metal sintering, but also needs to examine the performance of microwave receptor in improving the efficiency of converting microwave radiation into heat sources (Table 5).

**TABLE 5:** Effects of process variables on the catalytic performance for microwave-assisted DRM reaction with different catalysts.

Catalysts and microwave receptors	Temperature (^o^C)	CH_4_/CO_2_ ratio	Dielectric properties (tan δ)	CH_4_ conversion (%)	CO_2_ conversion (%)	H_2_/CO ratio	Ref
C-SiO_2_	650–900	1/1	>0.8	76–91	57–85	‒	Hamzehlouia et al. (2018)
5%Fe–C	900	1/1	<0.4	95	99	1.01	Li et al. (2019)
10%Fe–C	900	1/1	<0.4	98	100	1.02	
20%Fe–C	900	1/1	<0.4	93	100	0.93	
4%Fe/Al_2_O_3_-SiC	750	1/1	0.58	63	59	0.93	Zhang et al. (2018b)
8%Fe/Al_2_O_3_-SiC	750	1/1	0.58	87	88	0.96	
12%Fe/Al_2_O_3_-SiC	750	1/1	0.58	93	92	0.98	
16%Fe/Al_2_O_3_-SiC	750	1/1	0.58	89	90	0.96	
FY5+ eFe	800	1/1	<0.4	72	93	‒	Bermudez et al. (2012)
CQ + eFe	800	1/1	<0.4	42	70	‒	

At the universal level, the combination of microwave technology with catalysts confers several key benefits:

1. Reduced Apparent Activation Energy: A consistent observation across numerous studies is the significant lowering of the apparent activation energy for DRM under microwave irradiation compared to conventional heating (Li et al., 2021; Li et al., 2024). This phenomenon, attributed to localized “hot spots” and potential non-thermal effects, enables high reactant conversions at markedly lower bulk gas temperatures, offering a profound energetic advantage.
2. Synergistic Coke Management: The most transformative advantage is the altered role of carbon deposits. While coke formation remains inevitable, under microwave heating, carbon acts as an excellent microwave receptor. Instead of solely causing deactivation, the coke generates intense local heating that vigorously drives the gasification reaction (C + CO_2_ → 2CO). This creates an *in-situ* self-cleaning mechanism, where the problematic byproduct is continuously consumed, thereby significantly enhancing catalyst longevity and operational stability (Che et al., 2004; Menéndez et al., 2010; Odedairo et al., 2016; Zhang et al., 2023a).
3. Direct and Volumetric Heating: Microwave energy is deposited directly within the catalyst bed, overcoming heat and mass transfer limitations associated with conventional external heating. This leads to rapid heating rates, elimination of reactor wall overheating, and superior overall energy efficiency, as energy is used to heat the catalyst itself rather than the entire reactor system (Fidalgo et al., 2008; Gangurde et al., 2018; Pham et al., 2020; Wang Sourav et al., 2023).

### 4.1 Metal-based catalysts

The quest is to combine high catalytic activity with strong microwave absorption. Most conventional metal oxide supports (e.g., γ-Al_2_O_3_, SiO_2_) are microwave-transparent with low dielectric loss, meaning they cannot directly couple with microwave energy to generate heat efficiently. In microwave-assisted DRM, catalysts utilizing such supports rely on several indirect heating strategies.

#### 4.1.1 Heating via *in-situ* formed microwave receptors

During the DRM reaction, carbonaceous species (e.g., coke, carbon nanotubes, graphene) are inevitably deposited on the catalyst surface. While this coke deactivates catalysts under conventional heating, it becomes a crucial asset under microwave irradiation. Carbon is an excellent microwave receptor. Therefore, once a small amount of carbon is formed, it acts as a *in-situ* receptor, generating localized “hot spots” that provide the thermal energy required for the reaction to proceed on the adjacent metal sites supported on the oxide support. The catalytic performance then becomes coupled to the nature and amount of this carbon deposit.

**FIGURE 3:**
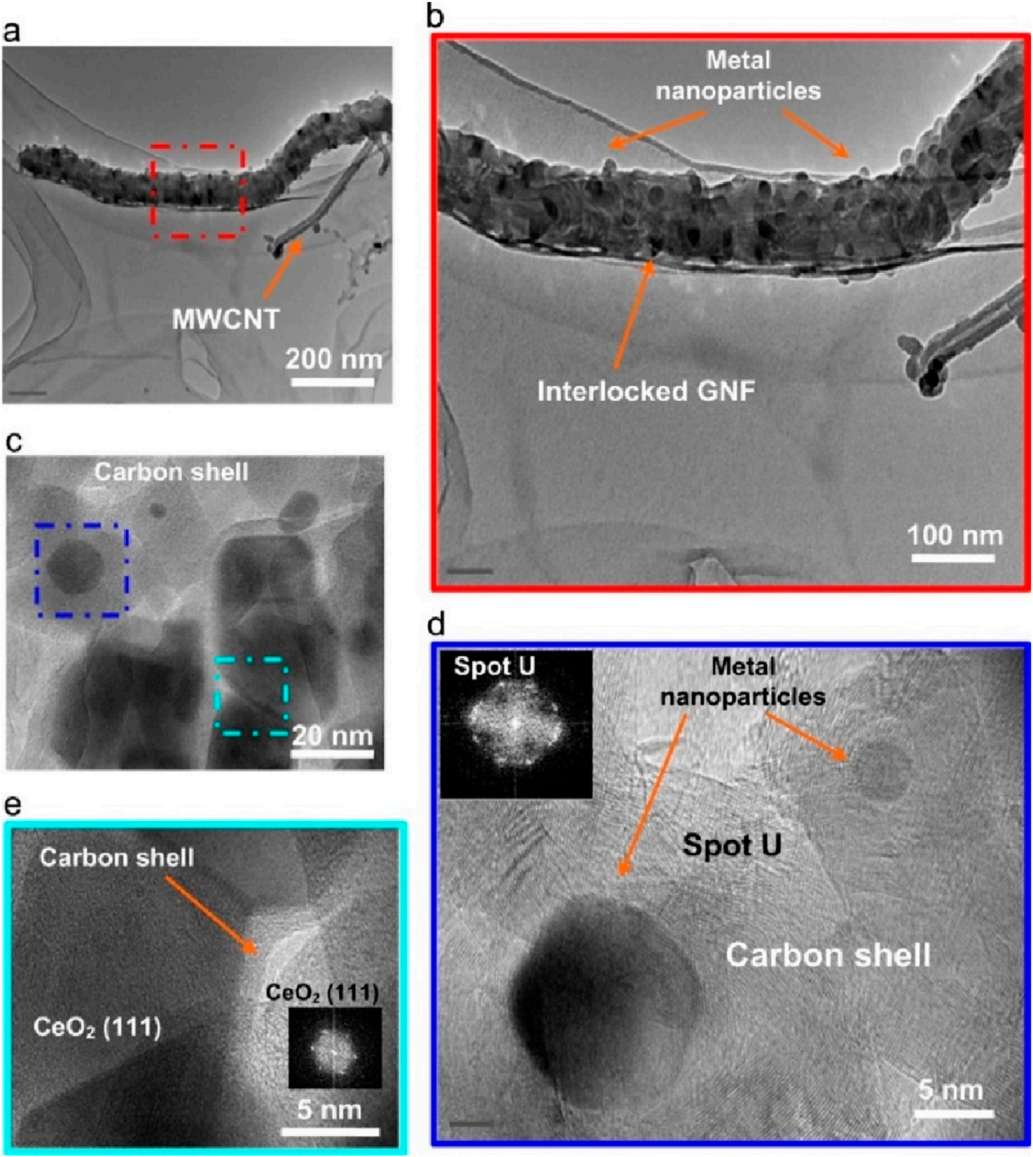
TEM micrographs with different scales **(a)** 200 nm, **(b)** 100 nm, **(c)** 20 nm, **(d,e)** 5 nm of multiwalled carbon nanotubes/graphitic nanofiber (M/GNF) formed on Ni/CeO_2_ after 14 h. Adapted from Ref (Odedairo et al., 2016).

Odedairo et al. investigated the structure of Ni/CeO_2_ catalyst with doping of Cr, Fe and Ta and the catalytic activity of the catalysts under microwave irradiation in dry reforming of methane was tested in a microwave reactor (Odedairo et al., 2016). The results show that the introduction of Cr and Ta to Ni/CeO_2_ can enhance the interaction between Ni and the support/promoter and inhibit the enlargement of NiO particles during the synthesis. The CH_4_ conversions in dry reforming on the catalysts follow the order: Ni/CeO_2_<2Fe-Ni<2Ta-Ni<2Cr-Ni. The superior performance of 2Ta-Ni and 2Cr-Ni may be attributed to the locally-heated Ni particles caused by the strong microwave absorption of the in situ grown graphene attached on them under microwave irradiation (Figure 3). The origin of this enhancement lies in the formation of microscopic “hot spots” on catalytic surfaces where local temperatures can be 100–300 K above the surrounding gas, accelerating both C–H and C=O bond scission (Fidalgo et al., 2008). Zhang et al. report efficient syngas production via methane reforming mediated by the microwave-triggered activation of Lanthanum nickelate (LNO) and carbon deposited on the catalysts could enhance the catalytic performance under microwave irradiation (Zhang et al., 2023a). It was observed that lanthanum oxycarbonates were active intermediates for CO_2_ activation and oxidation of carbonaceous intermediates from methane on the catalyst, thus improving catalytic activity and stability. This catalyst exhibited an advantage of process intensification and reaction stability in the microwave-assisted DRM process, in contrast to the conventional heating process.

**FIGURE 4:**
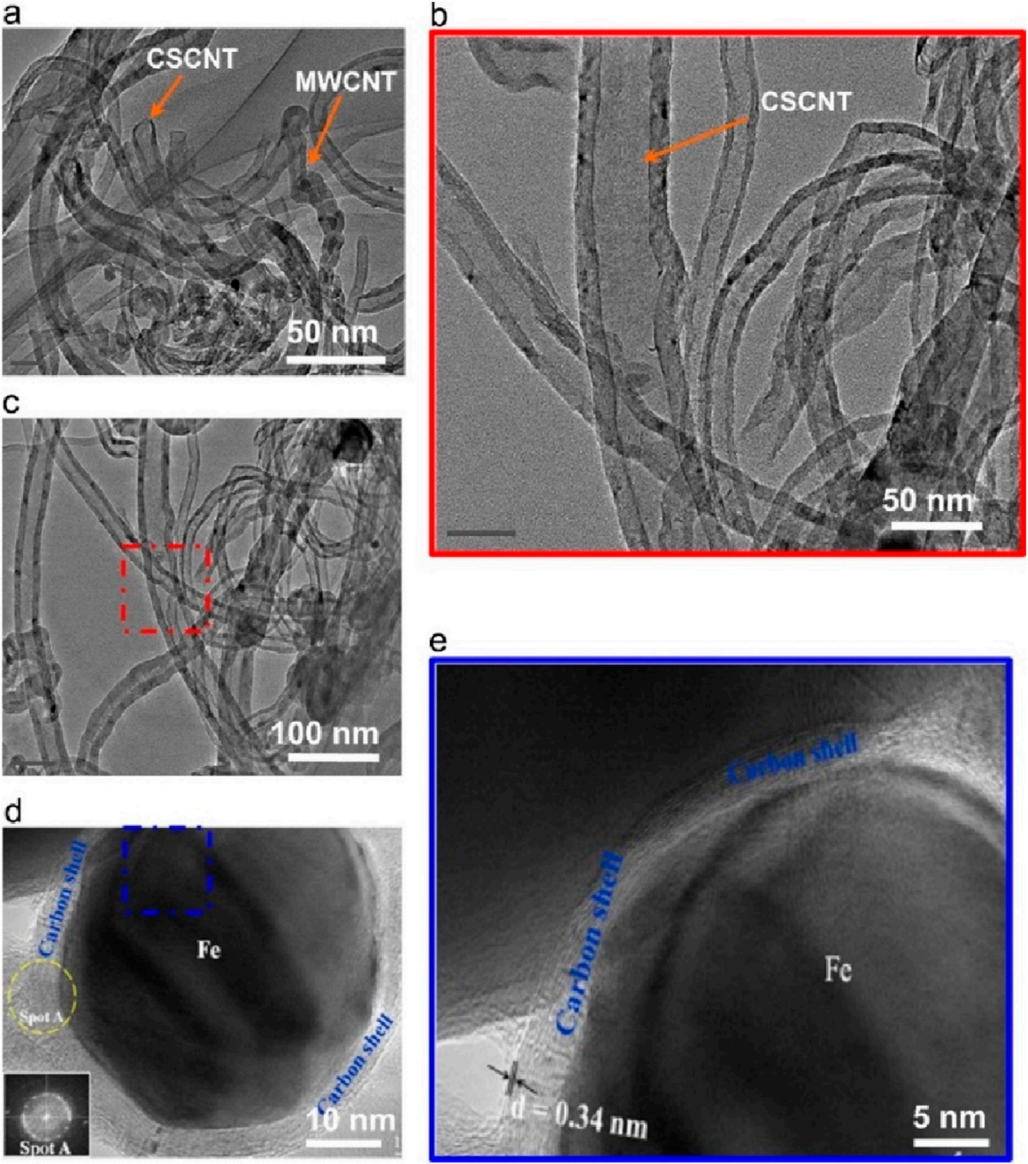
TEM micrographs with different scales **(a,b)** 50 nm, **(c)** 100 nm, **(d)** 10 nm, **(e)** 5 nm of multiwalled carbon nanotubes/cup-stacked CNT (M/CSCNT) formed on 2Fe-Ni after 14 h. Adapted from Ref (Odedairo et al., 2016).

In fact, the structure of carbon formed during microwave-assisted DRM also has a substantial impact on the reforming activity. Odedairo et al. found that carbon structure and morphology vary among the Ni catalysts promoted by several metals. Transmission electron microscopy (TEM) results confirme the presence of multiwalled carbon nanotubes/graphitic nanofiber composite (M/GNF) on Ni/CeO2 (Figure 4), while multiwalled carbon nanotubes/layered graphene composite (M/GR) on 2Cr-Ni and multiwalled carbon nanotubes/cup-stacked CNT composite (M/CSCNT) on 2Fe-Ni. And Carboncoated Fe nanocapsules are observed on 2Fe-Ni, which presented excellent microwave absorption properties (Che et al., 2004; Odedairo et al., 2016). Previous studies have reported that graphene owns plentiful of *sp* ^*2*^ π electrons, which makes it conducive to effectively absorbing microwaves and converting the subsequently absorbed microwaves into micro-plasmas or hotspots. Therefore, the generation of graphene during the DRM process is beneficial to the improvement of performance (Menéndez et al., 2010).

**FIGURE 5:**
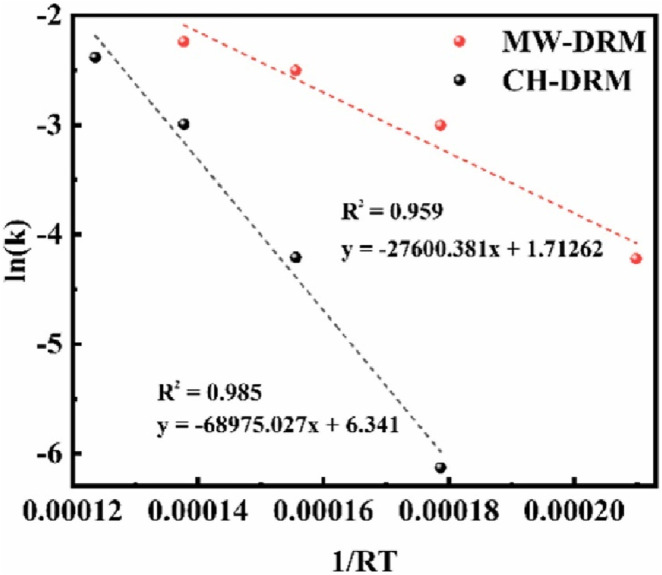
Activation energy fitting of CsRu/CeO_2_ under conventional heating (black) and microwave heating (red) conditions. Adapted from Ref (Li et al., 2024).

Kinetic analysis and empirical observations reveal that microwave-assisted DRM substantially reduces the activation energy required for the reaction, significantly enhancing the conversion rates of CH_4_ and CO_2_ into syngas (Li et al., 2024). Li et al. reported that compared to conventional heating dry reforming of methane (CH-DRM) processes, CsRu/CeO_2_ achieved higher methane (84.6%) and carbon dioxide (85.7%) conversion rates at a lower temperature of 500 °C in microwave-assisted DRM, significantly reducing energy consumption (Li et al., 2024). The significant decrease in activation energy for the microwave-assisted DRM process can be attributed to microwave-specific interactions with the CsRu/CeO_2_ catalyst (Figure 5). Microwave irradiation creates localized “hot spots” and enhances the electromagnetic field at the catalyst surface, likely reducing the energy barrier for CH_4_ and CO_2_ molecules to react (Li et al., 2021). This effect is further enhanced by structural and electronic modifications of the catalyst induced by microwave irradiation, promoting more efficient reactant conversion at lower temperatures.

#### 4.1.2 Heating via magnetic metal components

**FIGURE 6:**
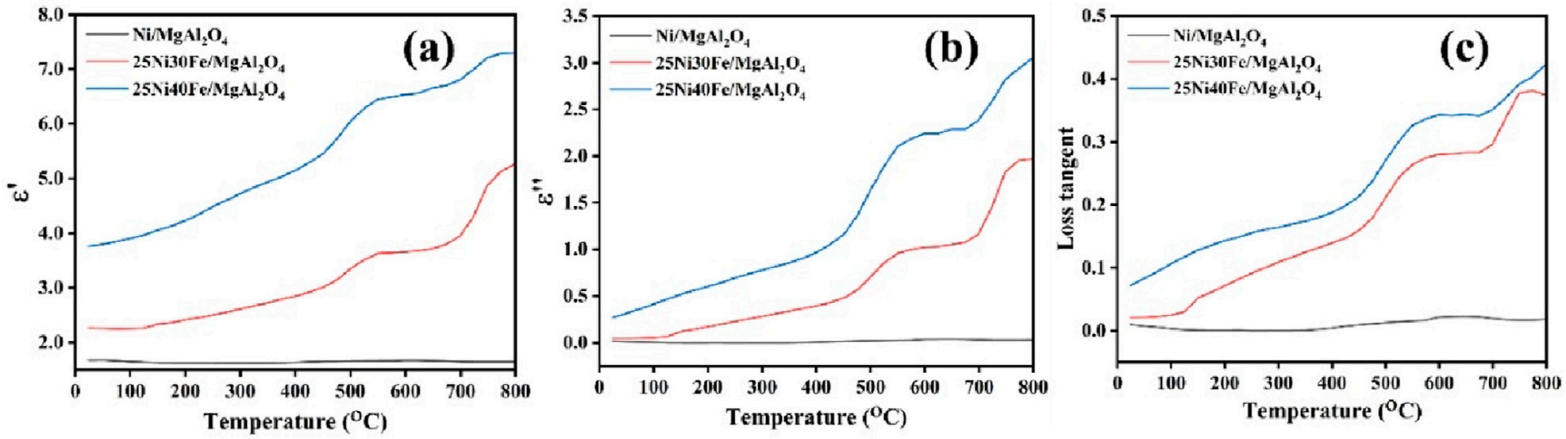
The temperature- dependent variation of **(a)** ε′(dielectric constant), **(b)** ε″ (dielectric loss factor), and **(c)** loss tangent for the Ni/MgAl_2_O_4_, 25Ni30Fe/MgAl_2_O_4_, and 25Ni40Fe/MgAl_2_O_4_ catalysts. Adapted from Ref (Olowoyo and Zheng, 2024).

The introduction of other metals can also lead to an improvement in the microwave-assisted DRM performance. Some active metals or promoters (e.g., Fe, Co., Ni) possess magnetic properties. Under microwave irradiation, these components can generate heat through magnetic loss mechanisms, effectively turning the metal nanoparticles into localized heaters. This heat is then transferred to the oxide support and the reaction site. Previous research revealed the crucial role played by Fe species in imparting microwave receptivity to the alloy catalysts, thereby significantly impacting DRM activity. Olowoyo et al. explored the effects of both microwave power and the temperature-responsive behavior of the catalysts, as well as delving into the influence of the active component content (Ni and Fe) and space velocity on DRM reactions (Olowoyo and Zheng, 2024). And found that the 25Ni40Fe/MgAl_2_O_4_ catalyst has a uniform distribution of Ni and Fe, the important role of iron incorporation in the structure of catalyst to increase its microwave absorption capability. (Figure 6) Zhang et al. evaluated the factors influencing the catalytic performance of Al_2_O_3_-SiC supported catalysts in the microwave-assisted DRM, including the catalyst carrier, microwave power, active component content, and space velocity (Zhang et al., 2018b). They found that an increase in the content of active Fe (4-12 wt%) could rapidly enhance the catalytic activity. However, when the Fe content reached 16 wt%, the catalytic activity decreased. When the Fe content reached the optimal value (12 wt%), the conversion rates of CO_2_ and CH_4_ reached approximately 92% and 93% respectively. When the content of active Fe was lower than 12 wt%, the active Fe was insufficient to be evenly distributed on the entire surface of the carrier, thus unable to provide sufficient active sites for the microwave-assisted DRM reaction, while a content of iron higher than 12 wt% was prone to cause metal sintering at high temperatures.

The magnetic properties of Co. particles led to a higher microwave absorption ability and exhibited better microwave-assisted DRM activity. Nguyen et al. reports on M-Mo bimetallic catalysts (M = Co. or Cu) supported on TiO_2_ for DRM under microwave irradiation (Nguyen Pham et al., 2020). Experimental results displayed outstanding activity of such M-Mo/TiO_2_ catalysts, on which high reaction efficiency of methane reforming can be sustained at a much lower microwave-assisted power of 100 W compared to literature results of 200 W. For DRM, about 81% CH_4_ and 86% CO_2_ were converted to syngas with a H_2_/CO ratio of 0.9 over the CoMo1 catalyst while the CuMo1 catalyst translated 76% CH_4_ and 62% CO_2_ into syngas with a H_2_/CO ratio of 0.8. The reason behind the excellent performance of the Co-Mo/TiO_2_ catalyst is the good exposure of the well-defined hexagonal microwave-assisted receptor. Meanwhile, the formation of a high dielectric layer of MoO_2_ surrounding active Cu^0^ can promote the microwave-assisted absorption of the Cu-Mo/TiO_2_ catalyst and thereby enhance its catalytic performance. The Co-Mo catalyst exhibited better activity than the Cu-Mo samples given that the magnetic properties of Co. particles led to a higher microwave-assisted absorption ability. Both M-Mo/TiO_2_ catalysts exhibited brilliant stability under microwave-assisted irradiation.

#### 4.1.3 Defect-driven microwave absorption in specialized oxides

**FIGURE 7:**
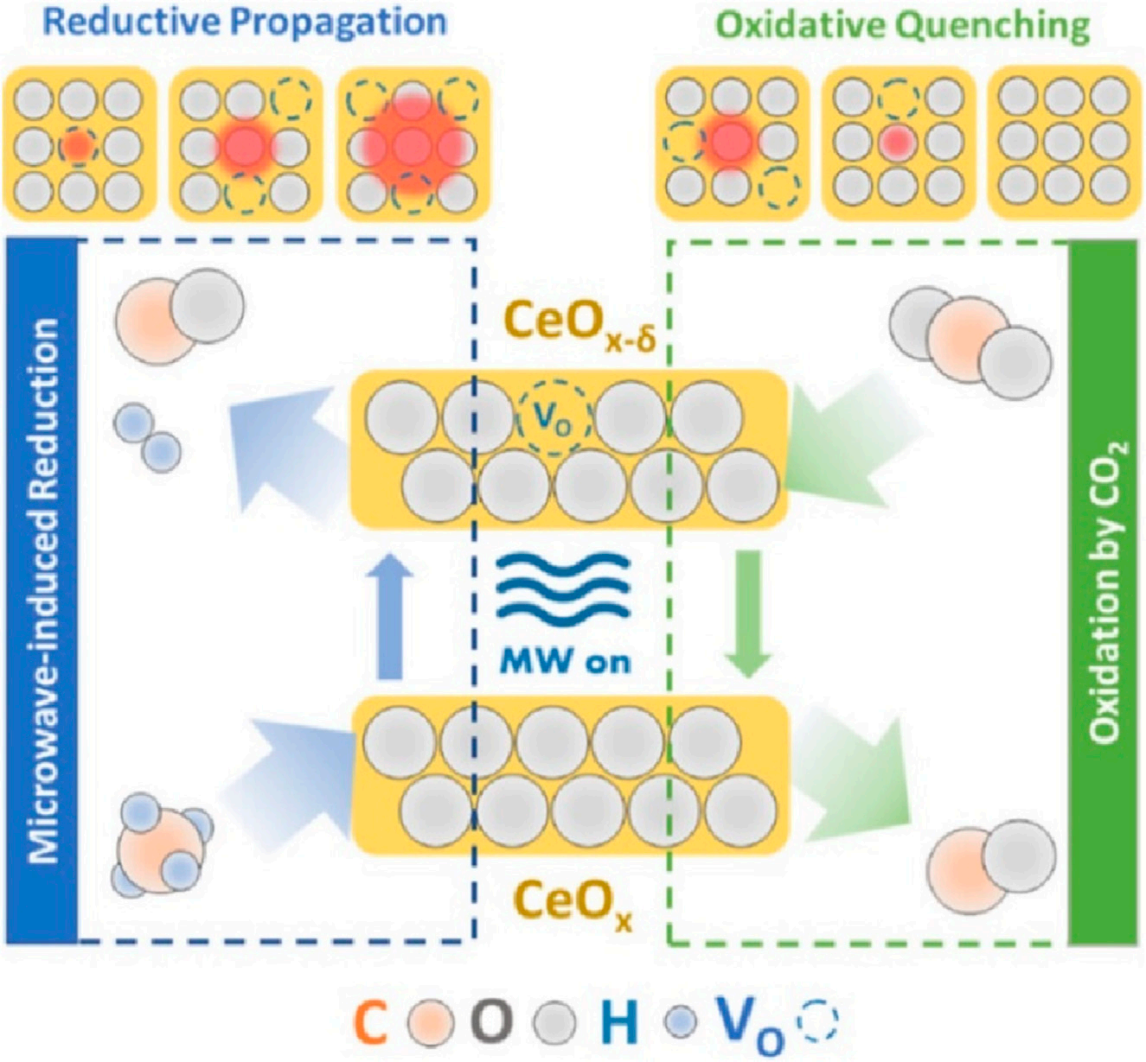
Schematic illustration of the oxidation/reduction Cycle. Adapted from Ref (Wang Sourav et al., 2023).

Furthermore, materials like Ce-Zr oxides can exhibit unique microwave-specific reaction pathways. Previous study revealed that Zr addition to ceria helps form Ce^3+^-V_O_ centers and suggesting the crucial role of these sites for the microwave-assisted DRM reaction. Wang et al. reported that the Ce^3+^-V_O_ pairs are reaction active sites and also the microwave absorbing sites, improving microwave heating and efficiency and propose an oxidation/reduction cycle (Figure 7) where a turnover invokes a Mars-van-Krevelen (MvK)-type chemical looping (Wang Sourav et al., 2023). The first half of the cycle is initiated by converting CH_4_ and O_L_ to CO and H_2_, forming an oxygen vacancy. The vacancy allows an adjacent lattice oxygen to hop in, rotating the imbalanced Ce^3+^-V_O_ dipole in the EM field and adsorbing energy to elevate the energy states at the nanoscale. This local excitation promotes additional conversion of CH_4_ and further reduction of the catalyst, creating a “reductive propagation” process. In the second half cycle, CO_2_ converts into a second CO molecule on the VO site and re-oxidizes the crystal. It effectively quenches the local energy states and creates a continuous single-bed periodic chemical loop. The reaction is periodically excited and quenched at the reactive centers without elevating the bulk measured temperature.

### 4.2 Non-metallic catalysts

**FIGURE 8:**
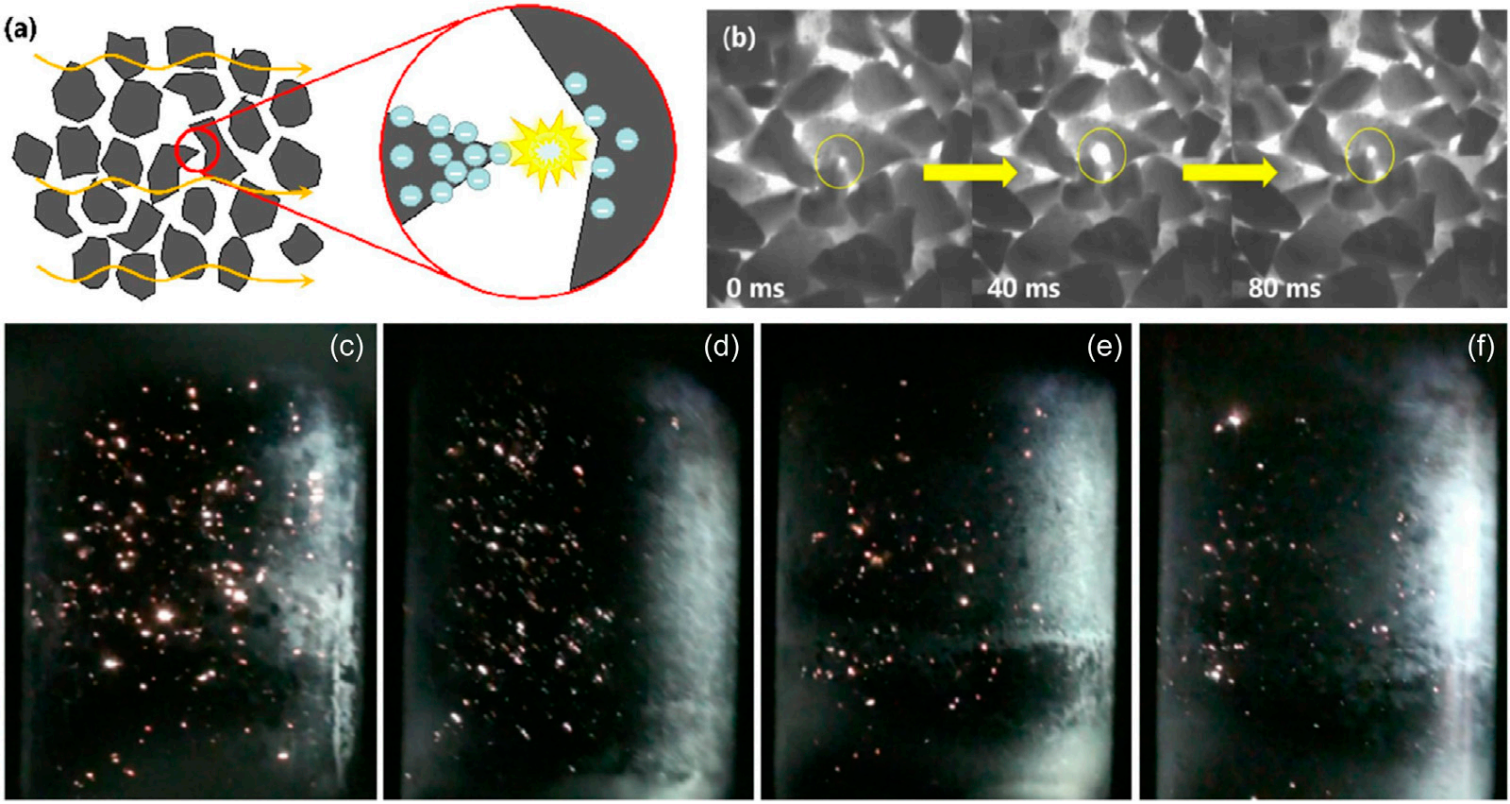
**(a,b)** Animated demonstration of charge accumulation and discharge evolution, **(c–f)** apparent phenomena of coconut shell activated carbon discharge with different particle sizes (20 s). Adapted from Ref (Zhang Zhang et al., 2023).

In addition to metals, carbon materials also discharge in the microwave field (Li Tao et al., 2020). When the carbon material is irradiated by microwave, the delocalized π electrons move freely in a relatively wide area. The kinetic energy of some electrons may increase, causing them to jump out of the material, which is thought to be a spark or arc. But at the micro-level, these hot spots are plasma (Menéndez et al., 2010). Compared with microwave-induced metal discharge, induced carbon material discharge has unique advantages in that it can generate plasma while maintaining intrinsic catalysis, and the material preparation cost is lower. Zhang et al. reported that the different carbon materials had different discharge intensities, but they all produced star-shaped sparks and particles with cis-fiber structure and tips placed in opposition to each other were easier to discharge in microwave electric fields (Figure 8) (Zhang Zhang et al., 2023). In addition, multi-point and frequent discharges are beneficial to improve the reforming effect. Based on similar catalyst structures, the enhancement of the discharge fluctuation improved the reaction effect more than that of increasing the temperature. And they revealed the mechanism of microwave discharge promoting the reaction process can be attributed to the synergistic effect of surface thermal catalysis and microwave plasma catalysis.

During microwave discharge, approximately 10% of the electron energy is utilized for electron excitation, while the remainder is allocated to vibrational excitation (Snoeckx and Bogaerts, 2017). As a result, vibrationally excited molecules play a critical role in plasma-catalysis reactions. Due to the relatively low electron energy in microwave discharge plasma, there is insufficient energy (>7 eV) to dissociate CO_2_ molecules directly into CO and O via electron collisions (Snoeckx and Bogaerts, 2017). In contrast, vibrationally excited CO_2_* species can persist for 10–100 μs and participate in chemical reactions (Liu and Chen, 2020). Similarly, microwave discharge generates a substantial amount of vibrationally excited CH_4_*, which also contributes to the reaction. Given that the C–H bond energy is lower than that of the C=O bond, CH_4_ fragmentation via electron collisions occurs more readily compared to CO_2_ (Khoja et al., 2019; Snoeckx et al., 2013). These collisions result in the formation of CH_x_ radicals and atomic hydrogen. Zhang et al. detected C and CH* peaks in the microwave discharge spectrum of a carbon catalyst, confirming that discharge excited CH_4_ and led to electron collision dissociation producing CH_x_ radicals, thereby facilitating the reaction process (Zhang Zhang et al., 2023). With the assistance of plasma, the adsorption and dissociation of CO_2_ and CH_4_ molecules on the catalyst surface are enhanced. Vibrational activation significantly increases the adsorption and dissociation probabilities of CO_2_* (Jiang and Guo, 2016) and CH_4_* (Fleming Crim, 2008), rendering them highly reactive. Meanwhile, CH_x_ radicals can also adsorb onto the catalyst surface and act as reactive intermediates in subsequent surface reactions (Nozaki and Okazaki, 2004), further promoting conversion.

Li et al. reported that bio-char catalytic activity could be affected by raw material for char preparation, since it was greatly connected to the remained metal after char preparation (Li Chen et al., 2018). In addition, original bio-char could only maintain its catalytic effect at an acceptable level in 70 min, due to an unavoidable carbon gasification reaction. Nevertheless, carbon gasification meanwhile generated part of CO production and it was a contributor to total syngas production. Tan et al. investigated the effect of activated carbon as catalyst on methane dry reforming and found that the wood-derived activated carbon exhibits better catalytic effect on methane reforming (Tan et al., 2019). Furthermore, they also calculated the migration amount of carbon and obtained carbon migration rate reduces with the increase of microwave power or decrease of CH_4_/CO_2_ ratio.

**FIGURE 9:**
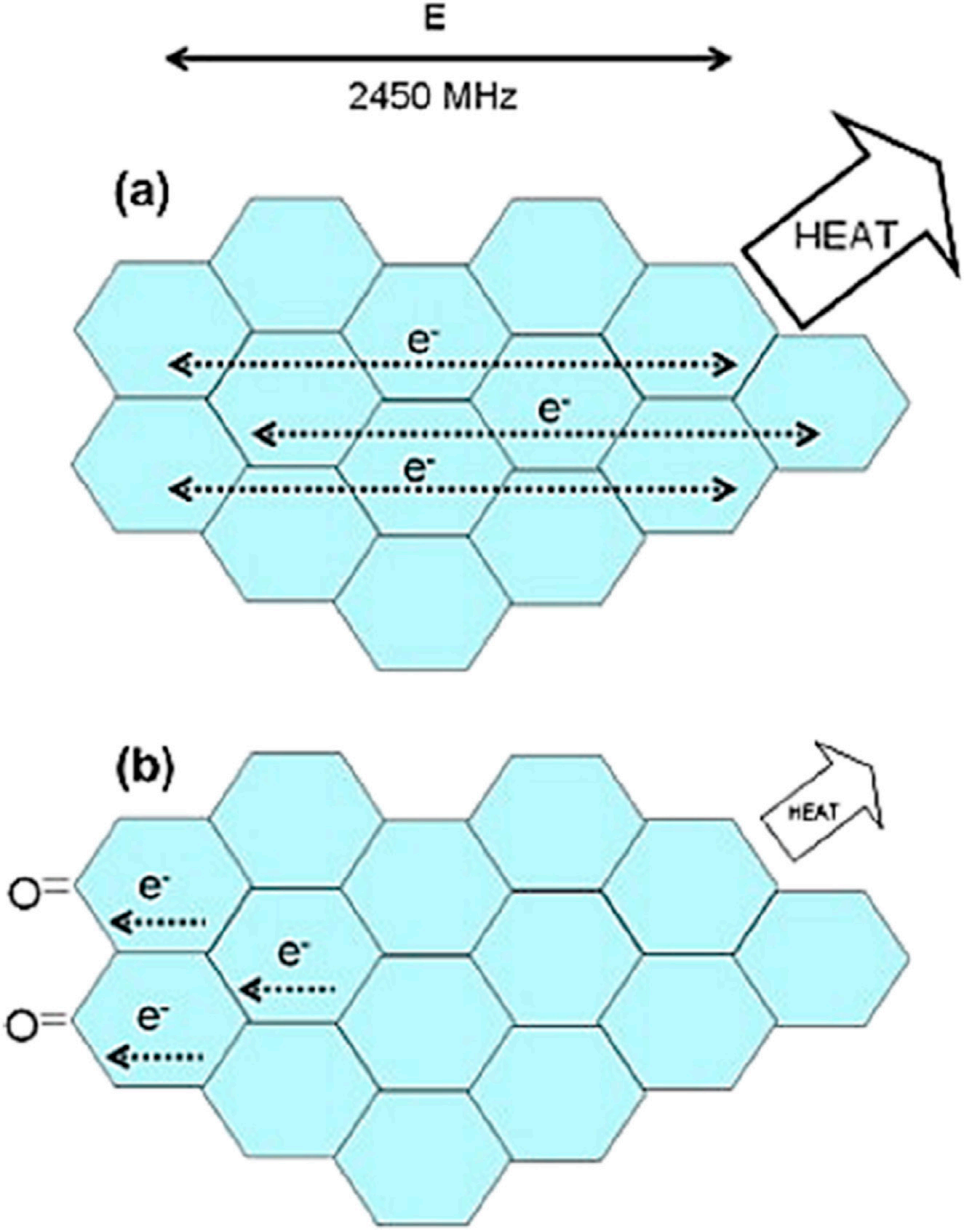
Schematic representation of some of the effects involved in the microwave heating of carbons: **(a)** When microwave heating is caused by the Maxwell-Wagner effect (Interfacial polarization), the delocalized π-electrons try to couple the changes of phase of the electric component of the electromagnetic field dissipating heat; **(b)** Hypothesis: oxygen-containing surface groups are electron-withdrawing, limiting the mobility of some of the π-electrons of the basal planes and therefore restricting the heat released. Adapted from Ref (Fidalgo et al., 2010).

Fidalgo et al. had synthesized various carbon materials with different textural and surface properties to investigate the influence of various carbon materials on microwave-assisted DRM performance and found that the dry reforming of CH_4_ occurs mainly in micropores and that, in addition to large micropore volume, the carbon material used as catalyst needs to show a good CO_2_ reactivity (Fidalgo et al., 2010). Thus, carbon materials with a low CO_2_ reactivity are not good catalysts for dry reforming reaction. Furthermore, the oxidized activated carbons are not the best catalysts for dry reforming, since the presence of oxygen surface groups reduces the catalytic activity of the carbon material dramatically, especially under microwave heating (Figure 9). The low conversions over oxidized carbons achieved in the microwave oven were due to their reduced CO_2_ reactivity and to the difficulty involved in heating them up.

### 4.3 Carbon-supported metal composite catalysts

The synergistic integration of carbon materials with active metals represents a cornerstone in the development of advanced catalysts for microwave-assisted DRM. This architecture is highly effective, leveraging the complementary properties of each component: the metal nanoparticles provide catalytic activity for CH_4_ and CO_2_ activation, while the carbon support acts as an efficient microwave receptor, enabling rapid and localized heating. This synergy is crucial for achieving high catalytic activity, stability, and product yields under the extreme conditions typical of DRM.

**FIGURE 10:**
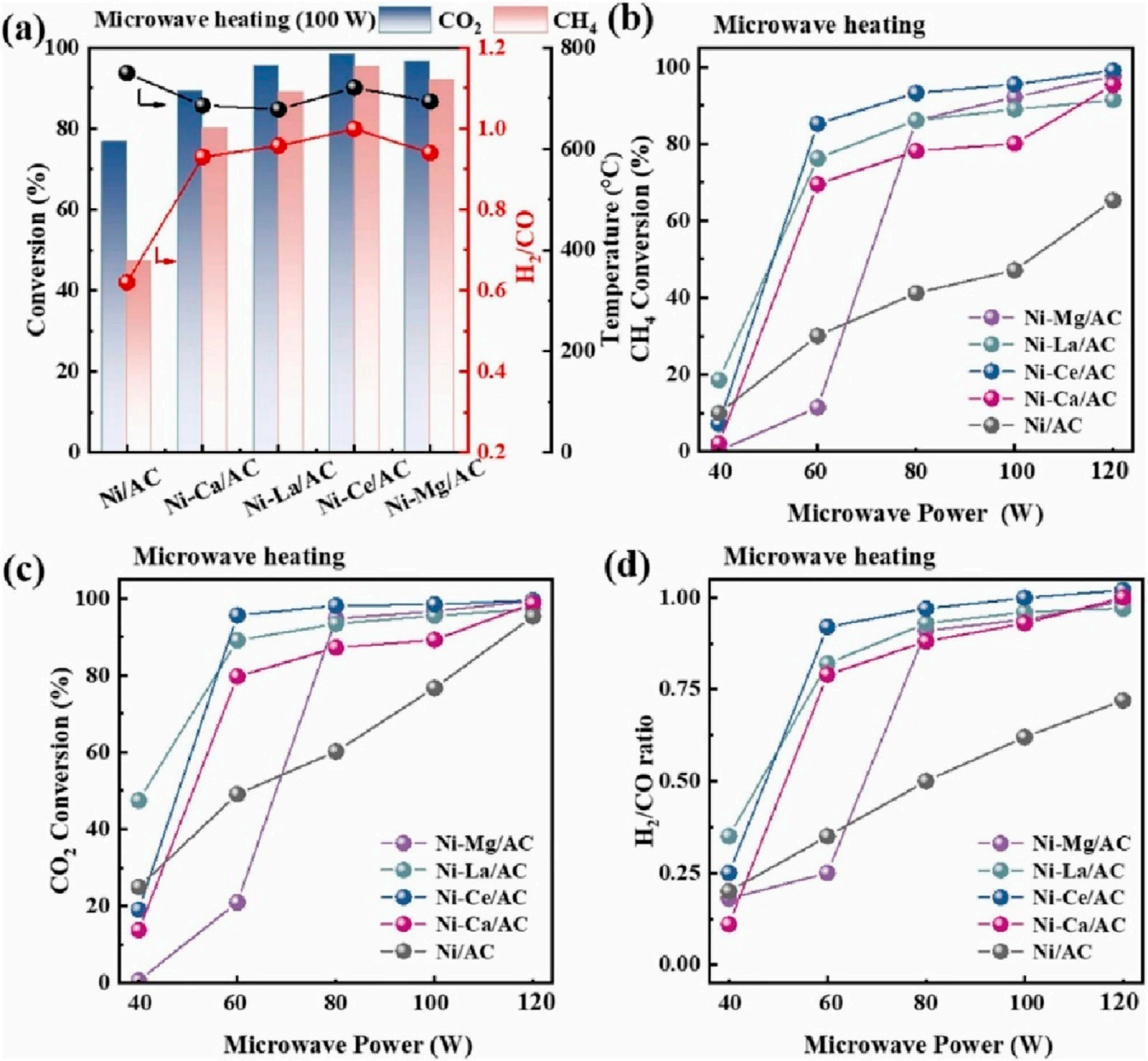
**(a)** Effect of **(a)** different promoters and **(b–d)** microwave power on reactant conversion rates and H_2_/CO ratio during microwave-assisted DRM process, (CH_4_: CO_2_: N_2_ = 1: 1: 6, Gas flowrate = 160 mL/min). Adapted from Ref (Zhang et al., 2025).

Zhang et al. prepared the activated carbon (AC)-supported Ni catalysts modified with Mg, Ca, La, Ce, and the Ni-Ce/AC catalyst was demonstrated the optimum catalyst for microwave-assisted DRM process (Figure 10) (Zhang et al., 2025). By converting microwave energy attenuation into heat and plasma, the locally-formed high-energy active sites composed of adjacent Ni, CeO_2_ and AC support of the Ni-Ce/AC catalyst could contribute to achieving the effective and localized activation of CH_4_ and CO_2_ molecules, thus leading to the enhancement of the reforming activity and the reduction of the loss of AC support due to CO_2_ gasification. Moreover, microwave heating method could avoid the excessive consumption of the AC support by increasing the graphitization degree of AC support and keeping a dynamic equilibrium state of the CH_4_ decomposition and CO_2_ gasification reaction, therefore contributing to the enhanced stability of the AC-based catalysts.

The surface engineering on the defects of the carbon-supported metal composite catalysts could strongly promote the electron transfer and improved the microwave absorption and conversion capacity and catalytic performance. Zhang et al. prepared the double-hierarchical Ni@C-NCNTs with different loading amount of defective N-doped carbon nanotubes (NCNTs) on the Ni@C microstructures, which significantly improved the microwave absorption and conversion capacity and catalytic performance due to the synergistic effect of multiple loss mechanism of the incident microwave and the mitigation of active metal sintering (Zhang et al., 2023c). Furthermore, Zhang et al. found the activation of lanthanum nickelates triggered by microwaves leads to the formation of lanthanum oxycarbonates, which were active intermediates for CO_2_ activation and oxidation of carbonaceous intermediates from methane on the Ni-La/AC catalyst, thus improving catalytic activity and stability (Zhang et al., 2023a).

Li et al. prepared the Ni/bio-char catalysts with varying Ni loading of 0–20 wt% to study the synergetic influence on their catalytic performance for microwave-assisted DRM at temperature of 800 °C (Li et al., 2017). The addition of Ni (from 0 to 20 wt%) could significantly enhance the bio-char’s catalytic performance and the optimal value was achieved at 10% Ni/bio-char respecting reactant conversions and catalytic stability. With the employment of Ni loading at 10 wt% and below, the equilibrium between carbon consumption and carbon deposition rate could be achieved, and hence, recovery of active sites led to higher CH_4_ conversion and better stability (Li et al., 2017). While the Ni/bio-char with Ni loading beyond 10 wt% experienced severe deactivation probably due to the active site blockage induced by metal sinterization and deposition of carbonaceous species, as evidenced by the post-reaction Fourier transform infrared spectroscopy and scanning electron microscopy measurements. It is worth noting that the conversion of CO_2_ was always greater than CH_4_ and the decrease in CO_2_ conversion was insignificant with growing Ni loading. This behaviour was possibly due to the gasification of carbon presented in bio-char and the reverse water–gas shift. From this study, Li et al. found that in the process of the carbon removal with CO_2_, the activeness of carbon from bio-char was much higher than carbon deposits in accordance with Fidalgo et al. (Fidalgo et al., 2008).

Apart from transition metal-based catalyst, Li et al. had prepared several alkaline metal-based catalysts and assessed their catalytic performance for microwave-assisted DRM (Li Chen et al., 2018; Li Yang et al., 2018). Interestingly, the advantages of employing alkaline metal were pronounced to the improvement in CO_2_ conversion, indicating the enhancement in gasification of carbonaceous deposits possibly owing to the extent of alkalinity of catalyst. In comparison with original bio-char, the embedment of K and Na improved the conversion of CO_2_ by 10.8% and 12.1%, respectively, while the embedment of Mg and Ca unexpectedly promote the rate of CH_4_ decomposition. Considering the economic feasibility, applicability and availability, Li et al. continuously study the microwave-assisted DRM process over the Fe-rich biomass-derived chars (Li et al., 2019). They found that Fe addition appreciably enhances the activity of methane reforming compared to original char. However, at Fe content beyond 10%, a drop in CH_4_ conversion was observed owing to the metal sintering at higher temperature. Conversely, the CO_2_ conversion was unaffected over different Fe content bio chars. This behaviour was attributed to the acceleration of both gasification of carbon and reverse water–gas shift contributing to the high consumption of CO_2_ reactant.

Above all, these findings provide new insights for the synergistic effect of microwave with carbon-supported metal composite catalysts for syngas production.

## 5 Catalyst performance comparison

**FIGURE 11:**
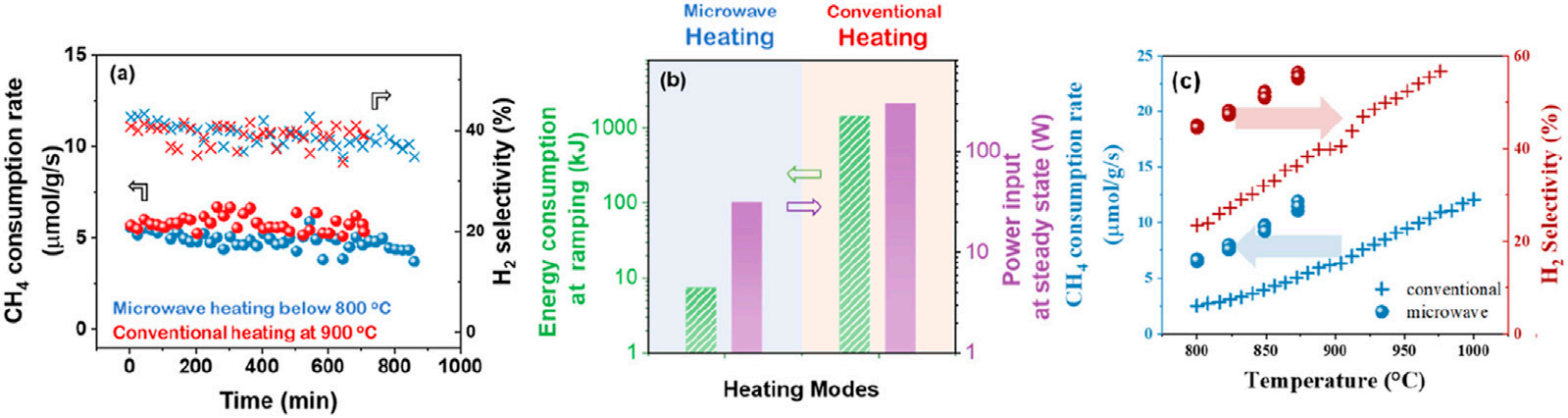
**(a)** Time-on-stream DRM reaction on ceria-zirconia catalyst in conventional heating and microwave heating at similar conversions. Reaction conditions: 100 cc/min total flow of 5% CH_4_ and 10% CO_2_ in N_2_. 250 mg catalyst loading, 900 °C in conventional heating, and ∼800 °C in microwave heating. **(b)** Energy consumption during temperature ramping and steady-state under conventional heating and microwave heating at similar conversions shown in **(a)**. Effect of temperature on the DRM reaction on the ceria-zirconia catalyst under microwave heating and conventional heating. **(c)** CH_4_ consumption rate (blue) and hydrogen selectivity (red) under microwave heating (●) and conventional heating (+). Adapted from Ref (Wang Sourav et al., 2023).

The comparative assessment on catalyst performance of conventional and microwave heating in DRM is seldom reviewed. It is evidently confirmed that microwave heating excelled in CH_4_-CO_2_ conversion performance compared to conventional furnace heating in terms of energy consumption and heating rates (Fidalgo et al., 2008; Gangurde et al., 2018). Hence, the microwave energy directly transfers to the microwave receptor/catalysts and enhances the internal temperature with precisely uniform distribution, eluding the energy wastage by heating the environs and reactor wall. However, a comprehensive comparison study of catalyst performance between conventional and microwave heating is yet to be done (Pham et al., 2020). Wang et al. were the first to measure core bed temperatures at high temperatures under microwave heating by using the fiber Bragg grating (FBG) temperature sensor, unachievable by pyrometers and IR cameras, enables a direct comparison with the conventional heating (Wang Sourav et al., 2023). And found that the reaction temperature under microwave heating reaches a steady state within 4 min, compared to ∼1.5 h in conventional heating. Importantly, microwave heating consumes less energy than conventional heating at these similar conversions: ∼150× lower (using 10 °C/min ramping rate in conventional heating) during temperature ramping and ∼10× lower at steady state, due to rapid and selective heating on the microwave heating susceptible catalyst. For the reactivity of ceria zirconia catalysts under microwave heating and conventional heating, the CH_4_ consumption rate and H_2_ selectivity increase with increasing temperature and are superior in microwave heating to conventional heating (Figure 11). The higher activity under microwave heating suggests that the density or turnover frequency of the active sites under microwave heating is higher.

**FIGURE 12:**
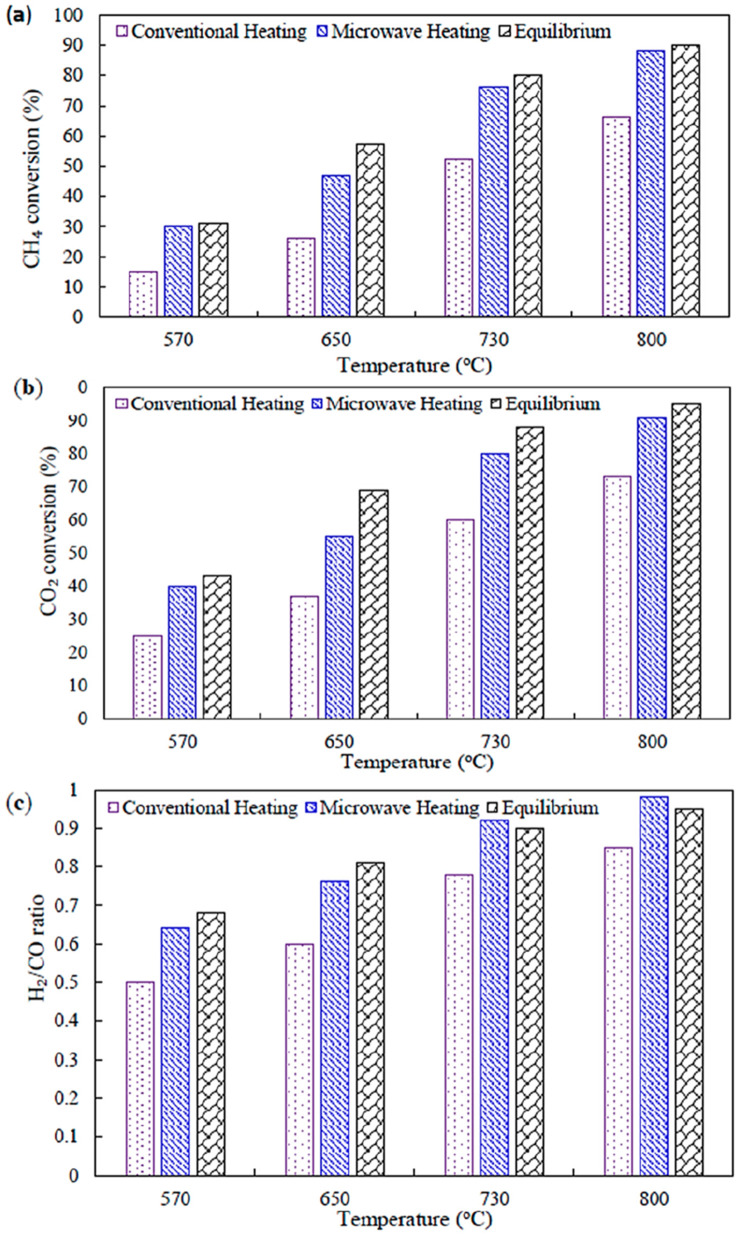
Comparison of microwave and conventional heating and equilibrium conditions for **(a)** CO_2_ and **(b)** CH_4_ conversions and **(c)** product selectivity as a function of temperature over Ni/MgO/AC catalyst and gas hourly space velocity (GHSV) of 33,000 mL/g cat. h. Adapted from Ref (Sharifvaghefi et al., 2019).

Sharifvaghefi et al. synthesized Ni-MgO/AC catalysts and compare the conversion, selectivity, thermal efficiency under convention heating and microwave heating (Sharifvaghefi et al., 2019). And found that the conversions of CO_2_ and CH_4_ were always higher under microwave conditions with an almost constant difference with conventional heating (Figure 12). In contrast to conventional heating, microwave heating can generate multiple hot spots at the catalyst surface, where local reaction temperatures are higher than the overall reaction temperature. In addition, with conventional heating, the H_2_/CO ratio was lower than the equilibrium values. In contrast, the H_2_/CO ratio was higher than the equilibrium value in the higher temperature range under microwave heating and it can be explained by the creation of hot spots, which is similar to that from the thermal temperature effect.

Table 6 shows the comparison of reported DRM catalysts under microwave-assisted system as well as conventional heating system. It can be seen that the conversion of CH_4_ and CO_2_ in the microwave-assisted heating system are generally higher than those in the conventional heating system in most catalysts, indicating that microwave heating promotes the catalytic performance of DRM.

**TABLE 6:** Comparison of reported DRM catalysts under microwave -assisted system as well as conventional heating system.

Catalysts	CH_4_:CO_2_	Temperature (◦C)	Microwave -assisted system		Conventional heating system		Ref.
			CH_4_/CO_2_ conversion (%)	H_2_/CO ratio	CH_4_/CO_2_ conversion (%)	H_2_/CO ratio	
10% Ni/Al_2_O_3_-F	0.5:1	700	72/46	N/A	42/32	0.75	Kustov et al. (2017)
10% Ni/Al_2_O_3_	0.5:1	700	61/53	N/A	42/30	0.53	Kustov et al. (2017)
5.6 wt% Ni/Al_2_O_3_ + FY5	1:1	800	90/80	N/A	80/55	N/A	Fidalgo et al. (2011)
Potassium-rich Char	1:1	800	90/98	0.7	70/97	0.7	Domínguez et al. (2007)
25Ni40Fe/MgAl_2_O_4_	1:2	700	85/62	∼1.0	N/A	N/A	Olowoyo and Zheng (2024)
Ni/MgO/AC (10% Ni and 10% MgO)	1:1	730	75/78	0.98	67/73	0.85	Sharifvaghefi et al. (2019)
10 wt% Fe/HZSM-5 + (BPC)	1:1	700	52/74	0.71	35/47	0.4	Zhang et al. (2022)
Active carbon (FY5)	1:1	800	60/53	N/A	80/55	N/A	Fidalgo et al. (2010)
Ni/Al_2_O_3_-m	1:1	650	N/A	N/A	63/70	0.9	Ma et al. (2021)
Ni-La/AC	1:1	700	99.8/99.8	1.01	N/A	N/A	Zhang et al. (2023a)
Ni/Al_2_O_3_+AC	1:1	800	88.1/93.3	N/A	N/A	N/A	Fidalgo and Menéndez (2012)
Ru/SrTiO_3_	1:1	702	93.9/90	0.9	N/A	N/A	Gangurde et al. (2018)
Cr-Ni/CeO_2_	1:1	850	90/92	1.45	N/A	N/A	Odedairo et al. (2016)
Ni-CeO_2_-Al_2_O_3_	1:1	800	N/A	N/A	70/80	0.9	Ma et al. (2021)
Fe/SiC	1:1	750	93/92	0.98	N/A	N/A	Zhang et al. (2018a)
La_0.8_Sr_0.2_Co_0.9_Mn_0.1_O_3_	1:1	850	83/90	0.93	N/A	N/A	Marin et al. (2021)
Ni/MgAlSiO_x_	1:1	800	N/A	N/A	80/82	0.98	He et al. (2021)
Ni/AC	1:1	900	N/A	N/A	68/82	1.05	Li et al. (2020b)

*N/A = not available in the reference.

## 6 Current challenges and future perspective

**FIGURE 13:**
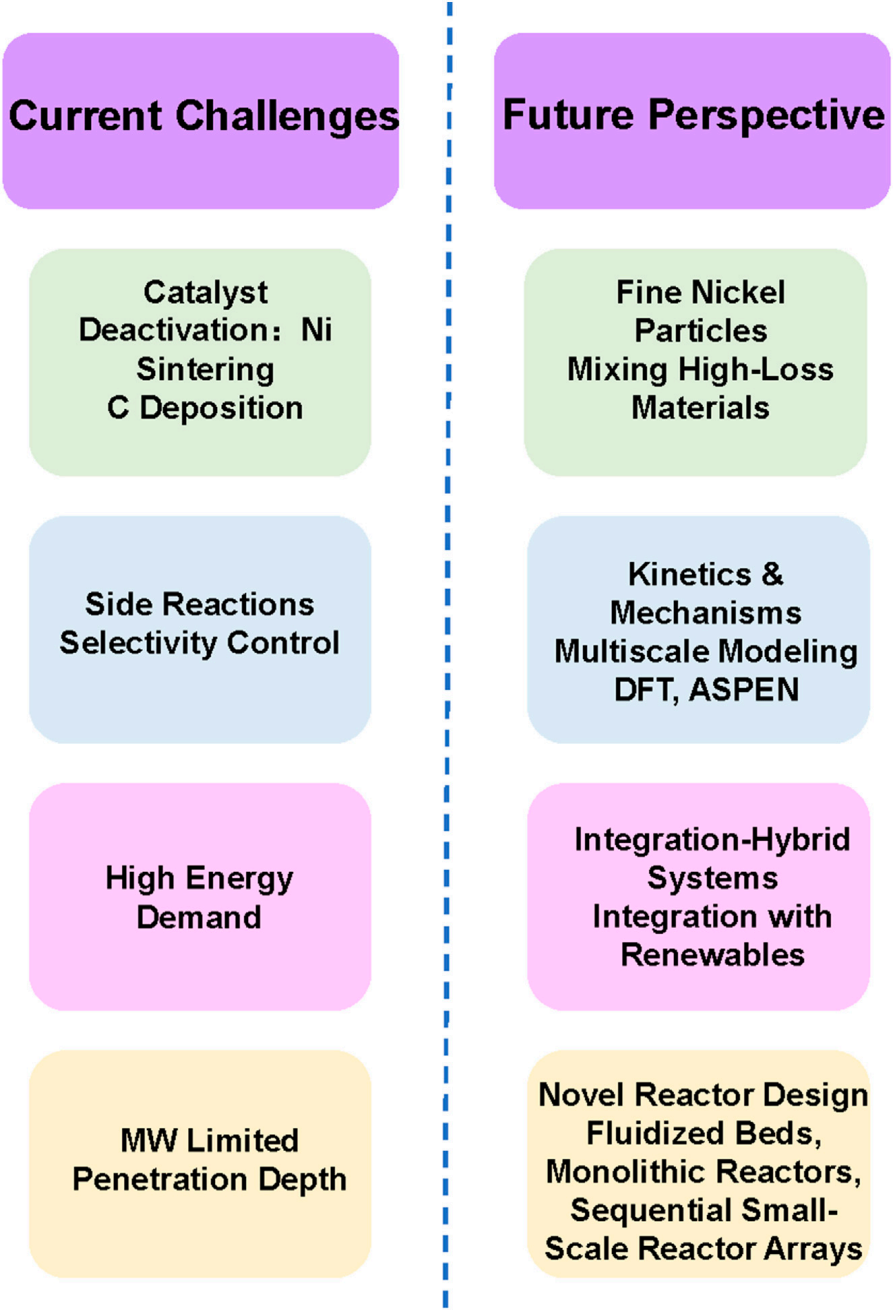
Current challenges and future perspective of microwave-assisted DRM.

Dry reforming of methane (DRM) holds significant environmental and energy sustainability implications as it converts greenhouse gases (CH_4_/CO_2_) into syngas (H_2_/CO), simultaneously addressing carbon neutrality objectives (Figure 13). The resultant syngas serves as a versatile feedstock for Fischer-Tropsch synthesis of energy-dense chemicals and hydrogenation processes for crude oil upgrading and methanol production, effectively reducing reliance on conventional fossil resources. However, technical challenges persist in mitigating Ni nanoparticle sintering and carbon deposition under high-temperature operating conditions (Bian et al., 2017). Moreover, the operating conditions (temperature, pressure, reactant ratio) may also lead to catalyst deactivation. For instance, an unbalanced reactant ratio or excessive steam content can promote carbon formation, accelerate catalyst deactivation, and cause the active sites of the catalyst to be blocked by carbon deposits, thereby inhibiting the adsorption of reactants. It leads to the reduction of catalytic activity and selectivity (Bian et al., 2017). Therefore, in order to solve this problem, researchers have studied the structural engineering of catalysts, such as developing structural catalysts with mixed metal oxides, zeolites, supported metals, *etc.*, to improve their stability and anti-carbon ability (Kawi et al., 2015).

Because the DRM process involves competitive reactions, such as the reforming of methane and the decomposition of carbon dioxide. Therefore, a thorough understanding of the kinetics and mechanism of the reaction is necessary to achieve selective control of the reaction path, maximize the selectivity of the desired product, and minimize by-products as much as possible. By studying the reaction intermediate and reaction pathways to optimize the process conditions, the overall efficiency of the DRM process can be improved (Han et al., 2020). Furthermore, due to the high dissociation energy of CH_4_ and CO_2_, they require a very high temperature for activation, and this reaction condition may lead to the deactivation of the catalyst (Jang et al., 2019). However, the current research lacks comprehensive thermodynamic or kinetic models as well as experimental data, which further hinders the progress in this field (Foppa et al., 2017). In addition, the carbon dioxide source of DRM usually comes from industrial flue gas, which contains impurities such as sulfur and may poison the catalyst, causing it to deactivate. Therefore, efficient carbon dioxide capture technologies are also needed to ensure high-quality raw material sources for the DRM process (Al‐Mamoori et al., 2017).

In response to the challenges faced by DRM in conventional heating system, the microwave-assisted DRM has improved both in terms of energy consumption and catalytic performance, and has given rise to promising results, thus deserved to be investigated deeply. However, there are still challenges in its industrial application, such as the penetration depth (Dp), at which microwave power decays to one/e of its surface value, imposes a strict constraint on reactor geometry. This limits the viable diameter of a fixed-bed reactor, as microwaves cannot uniformly heat large-scale beds, leading to significant temperature gradients and loss of process efficiency. Large multimode cavities require careful design to ensure even electric field distribution. Inconsistent coupling can cause arcing, localized overheating, and reactor damage. Preventing microwave leakage in large systems requires robust shielding and interlocking. Power scaling to megawatt levels also demands high-efficiency magnetrons and waveguides, which increase operational costs. The following are outlooks of microwave-assisted DRM:

1. Due to the high magnetic loss tangent value of Ni-based catalysts and their excellent catalytic performance in dry reforming of methane, more thoroughly research on them is necessary. Although metals are classified as microwave reflectors, as long as their particles are fine enough and the fineness is less than their penetration depth, they can be effectively heated by microwaves. Since the surface area of most microwave receptors is low, it is necessary to increase the number of fine nickel particles coated on microporous or mesoporous materials as much as possible, or by mixing high-loss materials (such as silicon carbide) with nickel-based catalysts to enhance its microwave absorption capacity (Shi et al., 2022; Shi et al., 2025).
2. A thorough understanding of the kinetics and mechanism of the reaction is necessary to achieve selective control of the reaction path, maximize the selectivity of the desired product, and minimize by-products as much as possible. By studying the reaction intermediate and reaction pathways to optimize the process conditions, the overall efficiency of the DRM process can be improved. There is a need to develop the thermodynamic equilibrium models which take into account the formation of hotspots or microplasmas to provide improved estimates of overall efficiencies (Li et al., 2021; Li et al., 2022; Marin et al., 2021; Rao et al., 2023; Shi et al., 2021; Shi et al., 2023; Wang Pu et al., 2023; Yabe et al., 2019; Zhao et al., 2022).
3. Integrating multiscale modeling approaches: Density functional theory (DFT) for mechanistic elucidation and catalyst interface optimization (Alotaibi et al., 2023; Khan et al., 2023; Yoon et al., 2022), machine learning algorithms for predictive catalyst screening and reaction parameter optimization (Artrith et al., 2020) and ASPEN for compare mass and energy balance of the conventional dry reforming of methane and microwave-assisted DRM (Almaraz et al., 2025). This theoretical framework enables rational catalyst design while minimizing empirical trial-and-error approaches, significantly accelerating developmental timelines. Particular emphasis should be placed on establishing structure-activity relationships for confined systems under non-equilibrium reaction conditions.
4. The industrial-scale implementation of microwave-assisted DRM necessitates sophisticated engineering solutions to preserve catalytic integrity (activity/stability/selectivity) alongside comprehensive techno-economic analyses encompassing capital/operational expenditures for commercial feasibility. Furthermore, multi-process integration strategies demonstrate enhanced system versatility through synergistic combination with complementary technologies. Furthermore, microwave-assisted DRM can also be integrated with steam reforming, Sabatier process, Fischer-Tropsch synthesis, CH_4_ synthesis and other processes, thereby obtaining a more flexible and universal production system. This system can simultaneously enhance carbon utilization efficiency, reduce greenhouse gas emissions, and prepare carbon-value-added chemicals. In addition, microwave-assisted DRM can also be integrated with renewable energy sources such as solar and wind power to enhance its sustainability and economic feasibility.
5. Novel reactor designs may be needed, moving away from traditional fixed beds toward fluidized beds, monolithic reactors, or sequential small-scale reactor arrays can mitigate penetration depth issues and improve heating uniformity. Multiphysics simulations combining electromagnetics, thermodynamics, and reaction kinetics can guide reactor optimization. Real-time adaptive control systems powered by AI may dynamically adjust power and gas flow to maintain stability.
6. A microwave-plasma hybrid system could uniquely address overcoming penetration depth limitations. A localized plasma torch, ignited and sustained by microwaves, acts as an intense, point-source of energy and active species. An array of such plasma sources could be strategically deployed within a large-scale reactor, effectively bypassing the penetration depth problem and ensuring uniform activation across the reactor volume. Furthermore, this synergy potentially leading to dramatic improvements in energy efficiency and providing novel pathways to suppress carbon deposition.
7. Shifting from continuous-wave to pulsed microwave operation opens new dimensions for probing reaction mechanisms and enhancing control. Applying short, high-power microwave pulses (µs to ms duration) allows for the decoupling of thermal and non-thermal effects. Time-resolved *in situ* characterization (e.g., transient DRIFTS, mass spectrometry) during and after a pulse can capture short-lived intermediate species and surface changes. This approach is powerful for unraveling the intrinsic reaction pathways and quantifying the specific role of the microwave field in lowering activation barriers. And pulsed operation enables sophisticated dynamic control strategies. By tailoring pulse frequency, duration, and power, it is possible to manage the rate of energy delivery to the catalyst surface. This can prevent localized overheating, allow for surface relaxation, and enable *in-situ* regeneration cycles to periodically remove carbon, thereby significantly enhancing catalyst longevity and process stability.

## 7 Conclusion

This review underscores the profound environmental and energetic significance of microwave-assisted DRM as a transformative technology for sustainable syngas production. By simultaneously utilizing two potent greenhouse gases, CH_4_ and CO_2_, microwave-assisted DRM directly contributes to carbon neutrality goals and offers a versatile route for producing value-added fuels and chemicals, thereby reducing dependence on conventional fossil resources. The transition from conventional thermal heating to microwave irradiation represents a paradigm shift in catalytic reaction engineering. This review has elaborated on the unique mechanisms through which microwaves enhance the DRM process. These features collectively lead to marked improvements in energy efficiency, catalytic activity, and stability.

Despite these promising advantages, the industrial implementation of microwave-assisted DRM faces multidisciplinary challenges encompassing reactor design, catalyst development, process scaling, and system integration. Overcoming limitations such as microwave penetration depth, electric field distribution, and the design of cost-effective high-power systems requires continued innovation and interdisciplinary collaboration. Looking forward, microwave-assisted DRM is poised to play a pivotal role in future sustainable chemical processes, especially when integrated with renewable energy sources and carbon capture technologies. By enabling more efficient conversion of greenhouse gases into synthetic fuels and chemicals, microwave-assisted DRM holds the potential to close the carbon cycle and facilitate a transition toward a circular economy. This review not only summarizes current advancements but also provides a roadmap for future research, emphasizing the need for integrated multiscale modeling, advanced reactor design, and hybrid process systems to realize the full potential of microwave catalysis in industrial applications.
